# Some Remarks Bearing upon Tuberculosis

**Published:** 1896-08

**Authors:** Cooper Curtice

**Affiliations:** Moravia, N. Y.


### THE JOURNAL OF COMPARATIVE MEDICINE AND VETERINARY ARCHIVES.

Vol. XVII. *AUGUST*, 1896. No. 8.

### SOME PERTINENT REMARKS BEARING UPON TUBERCULOSIS.

By COOPER CURTICE, M.D., D.V.S.,
MORAVIA, N. Y.

On August 29, 1893, the Guernsey herd of Mr. R. A. Borden, of Easton, N. Y., comprised fourteen head: seven cows, three bulls, two heifers, and two calves. Previously Mr. Borden had bought and lost by death a cow, Lady Champion, of a disease characterized by cheesy nodules in the lungs. The owner desiring to show a number at the State Fair, and noticing that his best one was ailing, called upon the State Board of Health to inspect them. An inspection made by Dr. Charles Mackey and the writer, who were detailed by Dr. Lewis Balch, the secretary, indicated that six cows and two bulls were tuberculous. The field notes of this inspection are in Table I.

On page 494 of Volume II. of the *Fourteenth Annual Report* (1893) *of the New York State Board of Health* occur the following passages, which sum up the discussion about this herd at that time, and the reasons for handling it in a manner different from that involving the complete annihilation of all suspected animals which seemed to be necessary heretofore. The passages are from a letter, an answer to another from Dr. Balch, and was the result of conversations and correspondence between Mr. Borden, Dr. Balch, and the writer.

"Another inspector" (Dr. Curtice), "writing under date of September 3, 1893, makes the following observation: 'The proposed methods of handling these cattle by slaughtering part and quarantining the others is by far the best that could be proposed. It was part of the scheme I had in mind when I wished that the Board of Health had its own farm for experimental purposes.'

"The scheme was not for us to propose to Mr. Borden, but now that he has accepted the burden of expense, and proposes to maintain strict quarantine, it seems well for us to accept. If Mr. Borden will hold out with his herd for two or three years or until the State has established an experimental farm, when the experiments could be continued, the Board of Health will have under its control one of the finest opportunities for ascertaining new facts regarding heredity that could be planned.

"I regard the proof that calves dropped from tuberculous dams, separated immediately and fed either on Pasteurized milk or healthy milk not sterilized, will not become tuberculous until exposed to the disease at an after-date, to be of the highest importance economically. . . .

"If by proper handling of these cattle we can såve their offspring to the State in a healthy condition we will have met a most serious question in a fortunate manner. To thus handle grade cattle easily, or comparatively easily, replaceable by others, is, on account of expense, clearly out of the question. In this instance the value of the cattle renders the experiment possible, even to be undertaken by the owner."

It was decided accordingly to kill four: the two-year old bull Champion, and cows Maid, Maud, and Elva, which stood next each other in the stable. Tuberculosis was detected in Champion, Maid, and Maud by physical examination alone. The other cattle gave no physical evidence of disease, and all but Maud were in fine order. She was inclined to be thin. Her condition was such, however, but a few months earlier for her to have been selected as a representative-Guernsey giving milk sufficient in quantity and butter-fats to enter the World's Fair butter-test at Chicago.

The yearling Angus was saved as the sire of the new herd, although the test proved him tuberculous. He represented the fruit of twelve years' careful selection on the part of Mr. Borden, and perhaps centuries on the part of all the breeders of the Guernseys. As Mr. Borden claimed, he seemed all that could be desired for a yearling.

The post-mortem examination of the slaughtered cattle on September 23d gave the following results:

*Champion.* In both testicles a number of cheesy nodules, some an inch in diameter. The left posterior pharyngeal lymphatic gland was filled with cheesy nodules. The right lung contained numerous caseous masses, one 3 inches in diameter. The cephalic lobe of the left was a solid mass of cheesy material. The posterior mediastinal gland was 8 inches by 3 by 2½. Another was 2½ inches in diameter; others were enlarged. The left bronchial was 2½ inches in diameter; all were filled with a soft caseous material. The intestinal lymphatics were studded with hemorrhagic points, and many had cheesy nodules. The portal glands were large and cheesy. In the liver-substance were a few scattered nodules. The lungs were discharging tubercular material through the trachea.

*Maid.* The left pubic gland was egg-size; both it and the right contained cheesy nodules. Glands along the trachea were enlarged and cheesy. Anterior mediastinal glands much enlarged, egg-sized. The posterior mediastinal glands were 8 inches by 6 by 3. Both lungs contained very large abscesses; these were old pleuritic adhesions. In the substance of the liver were numerous small tubercular foci. The portal gland was 2½ inches in diameter and cheesy. The spleen and omentum had numerous grape-like bodies attached, which extended into the pelvic cavity. Intestinal lymphatics presented numerous cheesy foci. The mucous membrane of the horns of the uterus were studded with numerous small cheesy foci.

*Maud.* The anterior lobe of the left lung was caseous; the median lobe and the right lung had many nut-sized foci. The large posterior mediastinal gland was 6 inches by 3 inches and cheesy; others were very large. The anterior mediastinals were also affected. The left bronchial was 2½ inches in diameter. The liver had a few cheesy nodules. The portal gland was enlarged and cheesy. There were tubercular masses in the intestinal lymphatic glands. Grape-like masses were on the omentum.

*Elva.* A three-inch mass was found in the right lung, and a small half-inch nodule in the cephalic lobe of the left. The posterior mediastinal was calcareous and cheesy. The left bronchial was 3 inches in diameter.

All were tuberculous, Elva, the least affected, might have been reserved with the others, but was selected to demonstrate the fact that those reserved were not so badly affected as those already slaughtered. The diagnosis of extent of disease was made upon the delayed reaction and general good physical appearance.

Mr. Borden placed the remaining animals which had reacted to the test in another barn, and after disinfecting the barn where all had been kept placed the healthy ones in it.

Dr. Mackey, a month or two later, returned and retested some of the cattle. The old bull Glenwood reacted, and was slaughtered. The doctor informs me that he found in one of the lungs but one quite thoroughly encapsulated nut-sized mass with a cheesy centre.

At the time of the sudden stoppage of work by the Board of

Health on the 31st of March, 1894, Mr. Borden's cattle were, with others throughout the State, released from quarantine, and the valuable experiment abandoned.

Mr. Borden, however, scrupulously continued it, and on the 19th of May retested the new herd. Of this test Mr. Borden writes me as follows: "I send you a record of the temperatures taken before and after injections. The cattle are all in good condition except No. 10, a bull calf, sired by the young bull Angus, which you condemned nearly two years ago. Nos. 11, 12, 13, 14, and 15 are also young calves sired by him. No. 15 is out of Rosalind, condemned at that time. The calves are two or three months old. No. 16 is Rosalind and No. 17 is Angus; Nos. 1, 3, 4, 5, and 7 are the cows that bore the other calves. The bull Angus and cow Rosalind seem to be well except as shown by the tuberculin-test. The other two cows you condemned run down so last winter that I killed them. It is a source of great satisfaction to me to have tested the cattle and have them come out as I think they have. It seems now as if it was safe to use the bull, as I have been doing for two years. Can his produce be any more subject to the disease now than if he were sound?"

The last paragraph refers to the feeling of doubt that Mr. Borden has had in reference to the breeding from Angus. The old superstition regarding heredity of tuberculosis by breeding is so engrafted in literature that it will be a long time before men fully understand that the disease is transmitted by animals living together and not by their breeding together. The disease has not yet destroyed the vitality of the young bull, and so long as he retains apparent good health so long will he be able to get healthy offspring, more especially when the dams are healthy in every way. Even the progeny of the tuberculous pair Angus and Rosalind will be free from tuberculosis until the disease is contracted just exactly as another animal of healthy parents contracts it, and will succumb to its ravages no sooner, no later.

Table II., herewith presented, shows that the only two affected cattle on the place are the two (Angus and Rosalind) found affected in August, 1893. The cattle that were killed last winter were Donna Juanna and Amberine. These cows were thought by Dr. Mackey and myself to have less of the disease at the time of examination than the others we killed. I have been asked by many how long the cattle we were killing would live if not killed. These cattle are a fair example. Those we slaughtered would not have survived through the winter of 1893 and 1894, save perhaps Elva. Two others did not survive the winter of 1894–1895. Two others are yet alive and seem healthy. If they had not been handled exactly as they have been all these tuberculous cattle would have spread disease among the younger cattle, and it would have been impossible for Mr. Borden to have conducted his business profitably from that time to this. As Mr. Borden states in his letter, he has been able to obtain six calves this year from his thoroughbred bull Angus. While there may be some infusion of new blood through the cows, he has practically built up a new herd on the remnants of the old, and preserved the strain of blood he was so long occupied in establishing.

The loss to Mr. Borden and the State, through tuberculosis causing death of cattle, through the slaughter and separation of affected cattle, and the loss of milk, has been great, but has been lessened by handling the herd in a conservative manner. The preservation of the blood of the old stock and building of the new herd on the same lines has been a positive saving of qualities it has taken years to gain.

The ruthless destruction of all the tuberculous cattle of the State will not only entail a dead loss in milk and beef, but, what is of more value, of years of time, labor, and thought that have been exercised in raising milch cattle to the high standard now attained.

In view of the success of this experiment and the larger one conducted on the same lines by Bang, of Copenhagen, should the eradication of tuberculosis from blooded herds not be carried on with more circumspection and an endeavor made to save strains noted for their inherent valuable qualities?

More method, more earnest thought is necessary in prosecuting warfare against this disease lest the remedy destroy the fruit of years of painstaking selection on the part of breeders. The men engaged in it should have other requisites than mere ability to inject tuberculin, register high temperatures, and wield the pole-axe: discrimination, in deciding upon ascertained facts, and judgment, to be used in the preservation of the valuable qualities for which many strains are noted, are necessary.

### Table I.—Registered Guernsey Herd of R. A. Borden.

Cattle injected at 10 P.M., August 29, 1893.

| | | | Before | After | | | | |
|---|---|---|---|---|---|---|---|---|
| | | | 9 P.M. | 8 A.M. | 10 A.M. | 12 M. | 2 P.M. | 3 P.M. |
| Mignonne of Castel, | 1,062 | 13 years. | 101.6 | 100 8 | 101.1 | 102.1 | 101 9 | |
| Donna Juanna, | 2,661 | 9 " | 101.6 | 100.6 | 102 3 | **104 2** | **105.8** | |
| Amberine, | 2,059 | 9 " | 101.4 | 101.4 | 102.2 | **104.0** | **105.4** | |
| Rosalind, | 48,294 | 7 " | 102 4 | 101.2 | 102.0 | **105.2** | **105.8** | |
| Maid, | 4,758 | 4 " | 103.6 | **107.3** | **106.9** | **104.1** | **105.0** | |
| Maud B. | 5,131 | 3 " | 103.6 | **107.3** | **107.0** | **107.4** | **105.6** | |
| Elva L. | 51,206 | 3 " | 101.2 | 101.8 | **103.9** | **105.7** | **105.5** | |
| Glenwood, | 531 | 10 " | 102.1 | 101.2 | 101.0 | 101.7 | 101.6 | |
| Champion, | 51,979 | 2 " | 104.1 | **105.8** | **106.6** | **107.4** | **108.0** | **107.2** |
| Angus, | | 1 " | 102.0 | 103.4 | **106.6** | **106.4** | **106.2** | |
| Belle of the Glen,[1] | | 1 " | 102.0 | 101.6 | 103.0 | 103.5 | 102.7 | 102.0 |
| Lady II.[2] | | 1 " | 101.8 | 101.7 | 100.8 | 100.8 | 101.3 | |
| Bull calf, | | 6 mos. | 102.5 | 101.5 | 101.6 | 101.6 | 101.8 | 101.5 |
| Heifer calf, | | 2 mos. | 103.0 | 103 2 | 102.6 | 102.6 | 102.9 | |

These cows were injected with Sibbertz's tuberculin, using 0.25 cubic centimetre as a dose for an adult.

### Table II.—Mr. Borden's New Herd Guernseys.

Injected Sibbertz's tuberculin, May 19, 1895.

| | | Before | | | | After | | | | | |
|---|---|---|---|---|---|---|---|---|---|---|---|
| | | 8 A.M. | 12 M. | 4 P.M. | 9 P.M. | 8 A.M. | 10 A.M. | 12 M. | 2 P.M. | 5 P.M. | 8 P.M. |
| 1 | Cow, | 101.5 | 102.2 | 101.3 | 100.8 | 101.2 | 101.4 | 101.2 | 101.3 | 101.0 | 101.0 |
| 2[3] | " | 101.0 | 102.5 | 102.3 | 102.2 | 102.6 | 102.6 | 102.5 | 102.8 | 102.6 | 102.8 |
| 3 | " | 101.6 | 102.6 | 101.2 | 101.9 | 101.4 | 101.7 | 100.7 | 101.2 | 100.5 | 101.2 |
| 4 | " | 101.0 | 100.6 | 100.8 | 101.0 | 100.7 | 100.2 | 101.0 | 101.0 | 100.6 | 100.8 |
| 5 | " | 101.6 | 101.0 | 101.4 | 100.5 | ..... | 101.4 | 101.2 | 101.4 | 101.3 | 100.2 |
| 6 | " | 101.7 | 101.2 | 101.1 | 101.4 | 101.7 | 102.1 | 102.0 | 102 0 | 101.6 | 102.4 |
| 7 | " | 101.3 | 100.3 | 99.2 | 101.6 | 101.2 | 101.4 | 100.7 | 101.6 | 101 0 | 101.2 |
| 8 | " | 102.3 | 102.0 | 101.8 | 101.1 | 101.4 | 101.6 | 101.0 | 101.2 | 101.0 | 101.1 |
| 9 | " | 102.3 | 102.1 | 101.3 | 101.0 | 101.8 | 101.7 | 101.8 | 101.0 | 101.0 | 101.8 |
| 10 | Calf, | 101.3 | 102.2 | 101.0 | 102.4 | 101.4 | 101 1 | 98.9 | 101.0 | 101.5 | 100.6 |
| 11 | " | 103.3 | 102.7 | 102.8 | 103.0 | 102 4 | 102.0 | 102.4 | 102 6 | 102.1 | 102.8 |
| 12 | " | 102.0 | 101.7 | 102.0 | 103 0 | 101.7 | 101.8 | 101.7 | 102.4 | 102.4 | 102.1 |
| 13 | " | 102.1 | 102.4 | 102.2 | 102.6 | 102.0 | 102.0 | 101.8 | 101.6 | 102.7 | 102.7 |
| 14 | " | 102.6 | 102.6 | 102.8 | 103.0 | 103.2 | 102.6 | 101.8 | 102.0 | 101.8 | 102 7 |
| 15 | " | 102 0 | 102.1 | 102 3 | 102.7 | 102.6 | 102.5 | 102.0 | 102.0 | 102.6 | 102 5 |
| | | | | | | | | | 1 P.M. | 4 P.M. | 6 P.M. |
| 16[4] | Cow, | 100.0 | 101.4 | 101.0 | 101.0 | 101.0 | 101.7 | .... | 103.2 | **106.0** | **105.5** |
| 17[5] | Bull, | 99.8 | 101.0 | 102.0 | 101.0 | 101.0 | 101.8 | .... | **105.0** | **104.5** | **104.5** |

[1] Rosalind's daughter. [2] Lady Champion's daughter.
[3] This cow was heavy with calf and calved three days afterward.
[4] Rosalind. [5] Angus.

**On to Buffalo!**

